# A Reverse Transcription-Polymerase Spiral Reaction (RT-PSR)-Based Rapid Coxsackievirus A16 Detection Method and Its Application in the Clinical Diagnosis of Hand, Foot, and Mouth Disease

**DOI:** 10.3389/fmicb.2020.00734

**Published:** 2020-05-12

**Authors:** Shiyu He, Yanzhi Huang, Yanling Zhao, Bo Pang, Lixue Wang, Liwei Sun, Haoyan Yu, Juan Wang, Juan Li, Xiuling Song, Hui Li

**Affiliations:** ^1^Department of Hygienic Inspection, School of Public Health, Jilin University, Changchun, China; ^2^Research Laboratory, Changchun Children’s Hospital, Changchun, China

**Keywords:** hand, foot, and mouth disease, Coxsackievirus A16, polymerase spiral reaction, isothermal nucleic acid testing, rapid diagnosis, onsite detection

## Abstract

Hand, foot, and mouth disease (HFMD) is a common viral illness affecting infants and children that is usually caused by Coxsackievirus A16 (CVA-16). To diagnose HFMD, we developed a method for rapid detection of CVA-16 based on reverse transcription-polymerase spiral reaction (RT-PSR). We used two pairs of primers that specifically recognize the conserved sequences of VP1 coding region of CVA-16, and template RNA was reverse transcribed and amplified in a single tube under isothermal conditions, total reaction time could be reduced to less than 40 min. The detection limit of this method was between 2.4 × 10^2^ and 2.4 × 10^1^ copies/μl with excellent specificity. To test the clinical applicability of the method, 40 clinical stool samples were analyzed using RT-PSR and quantitative reverse transcription-polymerase chain reaction, and comparison showed that the coincidence rate was 100%. Compared with other similar detection methods, RT-PSR requires less time, simpler operation, and lower cost. These results prove that our novel, simple, and reliable isothermal nucleic acid testing assay has potential application for clinical detection of CVA-16.

## Introduction

Hand, foot, and mouth disease (HFMD) has long been a focus of global public health, especially for children aged < 5 years (Huang et al., 2018; Qi et al., 2018). Enteroviruses (EVs), especially EV-A71 and Coxsackievirus A16 (CVA-16), are the main etiological agents involved in HFMD (Solomon et al., 2010; Chen et al., 2013). Since around 2014, CVA-10 and a new CVA-6 genotype have also become widespread (Chen et al., 2017; Broccolo et al., 2019). In the past few years, CVA-16 infection has caused several severe epidemics in Southeast Asia and some Asian countries (Liu et al., 2014; Koh et al., 2016; Rao et al., 2017), as well as some European countries (Ljubin-Sternak et al., 2011; Cabrerizo et al., 2014). In recent years, due to the widespread use of EV-A71 vaccine in China, the rate of HFMD caused by this strain has decreased significantly (Chong et al., 2012; Yi et al., 2017; Li Y. et al., 2019). Therefore, CVA-16 was identified as our research focus.

Hand, foot, and mouth disease is sometimes difficult to diagnose based on its clinical symptoms alone, as some symptoms are also common in other respiratory diseases. Since the last decade, researchers have been developing cost-effective virus detection techniques. To date, many investigations for CVA-16 detection have been established, including virus isolation combined with serological test, immunological, and molecular biological methods. The virus isolation combined with serological test is the gold standard for detection, but it is time-consuming and complicated to meet the requirements of prevention and rapid detection (Lin et al., 2008). Immunological methods such as colloidal gold immunochromatographic assays (CGIAs) have resolved the problem of being time-consuming, but they have poor accuracy (Zhang et al., 2016). Quantitative real-time fluorescent reverse transcription polymerase chain reaction (qRT-PCR) is a commonly used clinical screening method for HFMD and has excellent sensitivity and practicality but is still expensive and requires sophisticated instruments (WHO, 2011; Cui et al., 2013). To overcome these obstacles, it is important to establish a simple, economical, and visually identifiable CVA-16 detection method.

Polymerase spiral reaction (PSR) was developed by Liu et al. (2015). PSR combines the advantages of loop-mediated isothermal amplification (LAMP), without complicated heterothermic apparatus, and simple PCR primer design for rapid and effective detection of pathogens. Since its emergence, PSR has been applied to the detection of bacteria and double-stranded DNA viruses (Das et al., 2018; Malla et al., 2018; He et al., 2019). However, Coxsackievirus is a member of the Picornaviridae family of non-enveloped single-stranded RNA viruses, whose amplification involves a complex process of reverse transcription. In this study, reverse transcription and amplification were performed in a single system at a constant temperature simply by adding DNA polymerase and reverse transcriptase to the reaction system. In order to accelerate the reaction, we also designed accelerated primers so that the entire reaction could be completed within 40 min. Thus, a simple heating facility (such as a water bath) was sufficient to implement the RT-PSR requirements, eliminating costly thermal cycle instrumentation. By visualizing the turbidity change of the mixture, real-time fluorescence monitoring, and hydroxynaphthol blue (HNB) chromogenic dye, the RT-PSR results could be quickly visualized. Afterward, the results were confirmed by 1% agarose gel electrophoresis. RT-PSR provides strong technical support for real-time and on-site diagnostic, which fully proves its great potential in the screening and extensive application of HFMD pathogens.

## Materials and Methods

### Materials and Apparatus

Bst 2.0 WarmStart DNA polymerase (New England BioLabs, Ipswich, MA, United States) and AMV Reverse Transcriptase (Shanghai, China) were selected for RT-PSR. (NH4)_2_SO_4_, KCl, MgSO_4_, and NaCl were purchased from Sinopharm (Beijing, China). HNB and Tween 20 were purchased from Macklin (Shanghai, China) and DingGuo Changsheng (Beijing, China), respectively. Tris-HCl and betaine were obtained from Sigma (St. Louis, MO, United States). EvaGreen was obtained from YeSen (Shanghai, China). MonTrack safe red nucleic acid dye was purchased from Monad Biotech (Wuhan, China). Reverse transcription kit and dNTP mixture were purchased from TAKARA (Kusatsu, Japan). Diethylpyrocarbonate (DEPC)-treated water was purchased from Sangon Biotech (Shanghai, China). CVA-16, EV-A71, and CVA-6 One-step reverse transcription PCR kit was purchased from Daan (Guangzhou, China). Norwalk virus nucleic acid test kit was purchased from Land medical (Hubei, China). Rotavirus A colloidal gold detection kit was obtained from Wantai Biotech (Beijing, China). Fetal bovine serum and RPMI 1640 medium were purchased from Gibco (New York, United States). Tris-EDTA (TE) buffer solution (pH = 8) was home-made. The instrument used for real-time fluorescence quantification (LightCycler 96) was from Roche (Basel, Switzerland). NanoDrop2000 ultraviolet-visible spectrophotometer, biosafety cabinets, clean benches, and PCR thermocycle instrument were obtained from Thermo Fisher Scientific (New York, United States). Automatic nucleic acid extractor (Smart 32) was purchased from DaanGene (Guangzhou, China). Electrophoresis apparatus and digital gel imaging system were obtained from Tanon (Shanghai, China).

### Virus Culture and RNA Extraction

All the virus strains were from the Children’s Hospital of Changchun (China). Each virus used in this study underwent clinically standardized tests. Specimens identified as positive for CVA-16 by qRT-PCR were inoculated into African green monkey kidney (Vero) cells, cultured in a 37°C, 5% CO_2_ incubator. We observed the cytopathic effect (CPE) daily, harvested virus culture fluid when CPE reached + + + or + + + +, and stored it at −20°C. After three cycles of freezing and thawing, the cells burst and released the virus, which was then centrifuged (3,000 rpm, 5 min). Viral RNA was extracted from the supernatant using an automated nucleic acid extractor according to the instructions. Other viruses, including Norwalk virus genogroup II (NVG II), Rotavirus A (RVA), CVA-6, and EV-A71, were used as non-target samples.

### Design of Plasmids and Primers

RT-PSR primers adopt the design concept of mixed primers, that is, the primer includes two sequences that are sequentially combined with the target sequence, and can be folded into a U-like sequence during amplification. After RNA was reverse transcribed into cDNA, the primers were combined with the target sequence, and the steps of amplification, folding, and dissociation were repeated using the strand displacement and polymerase activity of Bst DNA Polymerase, and finally the nucleic acid sequence was amplified. VP1 is the variable capsid protein of CV-A16, which includes a major immunogenic site. It can be used to diagnose HFMD by detecting the conserved coding sequence of VP1 (GenBank Accession No. KT327162.1), which has been confirmed by a series of reliable molecular biology references (Li W. et al., 2019; Richter et al., 2019). After performing blast sequence alignment in NCBI, two sets of primers were designed using the conserved sequence of the VP1 coding region as the target sequence (He et al., 2019), including primary primers (FT and BT) and accelerated primers (LF and LB). The primary primers were used for binding and folding with the target sequence, and the accelerated primers were used to accelerate the reaction process and achieve rapid detection. An additional set of PCR primers was designed based on the sequence. These primer sequences were synthesized and HPLC-purified at Sangon Biotech. To determine the detection limit, 220-bp VP1 coding region containing the target sequence was cloned into pUC57 vectors. The detailed sequence information of the plasmid and primers are shown in Table 1.

**TABLE 1:** Oligonucleotides used for reverse transcription-polymerase spiral reaction (RT-PSR) detection of Coxsackievirus A16 (CVA-16).

Primer/plasmid name	Sequences
Primer 1	FT1 GATTCGTTTGCTTGGCAGACCGTAGCCAAACCCAATGGTGAG BT1 GATTCGTTTGCTTGGCAGACCGGGTGGGTCCGTCATTTTCAC LF1 ACATGTACTGCAGTAATTGGGG LB1 CCACCAACCCATCAGTGTTT
Primer 2	FT2 AAGTGGGTTTCGGAGCCCCTTAGCCAAACCCAATGGTAGG BT2 AAGTGGGTTTCGGAGCCCCTGGTGGGTCCGTCATTTTCAC LF2 CAACACACATCTAGTCTCAATGAGA LB2 TCAGCATCATTACAATGCCCAC
PCR primer	CVA-16-F ACATGCGCTTTGATGCTGAA CVA-16-R GGGGACTGACACTTGAGCT
The cloned part of VP1 coding sequence (plasmid)	agctgtttac ctacatgcgc tttgatgctg aattcacatt tgtcgtagcc aaacccaatg gtgagctagt cccccaatta ctgcagtaca tgtatgtccc gccaggggct ccgaaaccca cttccagaga ttcgtttgct tggcagaccg ccaccaaccc atcagtgttt gtgaaaatga cggacccacc agctcaagtg tcagtcccct tcatgtcacc

### Establishment and Optimization of Reverse Transcription-Polymerase Spiral Reaction

To establish the RT-PSR method, firstly, we reverse transcribed the template RNA into cDNA and performed a PCR to ensure availability of the cDNA. To determine whether a PSR occurred, cDNA was used as a template without reverse transcriptase. The reaction was carried out in a final mixture of 25 μl, which consisted of 20.0 mM Tris-HCl, 10.0 mM (NH4)_2_SO_4_, 10.0 mM KCl, 8.0 mM MgSO_4_, 0.1% Tween 20, 1% Triton X-100, 0.8M betaine, 1.4 mM dNTP (each), 8 U Bst DNA polymerase, 1.6 μM Ft and Bt, and 0.8 μM LF and LB. After 2-μl templates were added, the total volume was made up to 25 μl with DEPC-treated water. The entire reaction was performed in a Roche real-time PCR instrument. The reaction conditions were set to collect fluorescence signals every 45 s, for 99 cycles, and the reaction temperature was constant at 63°C. After verifying the feasibility of the PSR, 10 U reverse transcriptase was added to the system, and RNA was used directly as a template for RT-PSR amplification.

Since the temperature has a huge impact on the efficiency of RT-PSR amplification, we performed the reaction at different temperatures (62–66°C) and screened the optimal temperature of the reaction by the amplified fluorescence curve. As a metal ion indicator, HNB is usually used to achieve visual product detection based on the color change of the reaction solution under natural light (Goto et al., 2009). To make it easier to distinguish negative and positive results with the naked eye, the concentration of HNB was optimized. After the reaction, different volumes (2.5, 2.0, 1.5, and 1.0 μl) of HNB indicators with a mass fraction of 0.2% were added to four groups of negative and positive samples and screened to observe the color change.

### Sensitivity and Specificity of Reverse Transcription-Polymerase Spiral Reaction

To determine the analytical sensitivity of RT-PSR, the plasmid was diluted 10-fold with TE buffer (2.4 × 10^8^, 2.4 × 10^7^, 2.4 × 10^6^, 2.4 × 10^5^, 2.4 × 10^4^, 2.4 × 10^3^, 2.4 × 10^2^, and 2.4 × 10^1^ copies/μl). We added 2 μl of the diluted plasmid template to the reaction system. Because the plasmid was used as a template, the system for sensitivity detection did not contain reverse transcriptase. DEPC-treated water was used as the negative control (NC). The reaction conditions were 64°C, 45 s, 80 cycles. For comparison, the sensitivity of clinical qRT-PCR was also tested. The qRT-PCR system contained master buffer solution, Taq enzyme system, and reverse transcriptase system. Its reaction procedure was as follows: reverse transcription at 40°C for 25 min, initial denaturation at 94°C for 2 min, followed by 40 cycles of denaturation at 94°C for 3 min, 93°C for 15 s, and 55°C for 45 s. A Ct value of 34.8 was set as the critical point for its positivity.

For validation of specificity, each non-target virus was validated using a corresponding clinically standardized method. The colloidal gold diagnostic kit was used for the detection of RVA. CVA-6, EV-A71, and NVG II were tested using their qRT-PCR test kits, respectively. The nucleic acid of each virus was separately extracted and detected using RT-PSR. After elected samples that can be used for specific detection, each virus nucleic acid was separately extracted and detected using RT-PSR method. DEPC-treated water was used as the NC. The reaction conditions were 64°C, 45 s, 80 cycles, and the reaction system contained both polymerase and reverse transcriptase. After the reaction, all the amplification products were separated by 1% agarose gel electrophoresis, and HNB dye was added for visual identification. Unless noted otherwise, parallel control and NC were included in each run, and the results were reproducible.

### Validation With Clinical Samples

Forty clinical stool samples were randomly selected for examination. Normal saline (1 ml) was added to samples (1 g) and then subjected to percussion blending. Supernatant (200 μl) was absorbed and added to the nucleic acid extraction kit. Protease K (20 μl) was added to each sample for nucleic acid extraction. Since qRT-PCR is the most widely used virus nucleic acid detection technology for CVA-16 in clinical practice (Tan et al., 2008; Cui et al., 2013), we performed qRT-PCR and RT-PSR on the extracted RNA. The results of the two methods were compared. We also collected the case information of these patients such as age, length of stay, symptoms, and signs, and we performed further comparisons and analysis.

## Results

### Establishment and Optimization of Reverse Transcription-Polymerase Spiral Reaction

**FIGURE 1:**
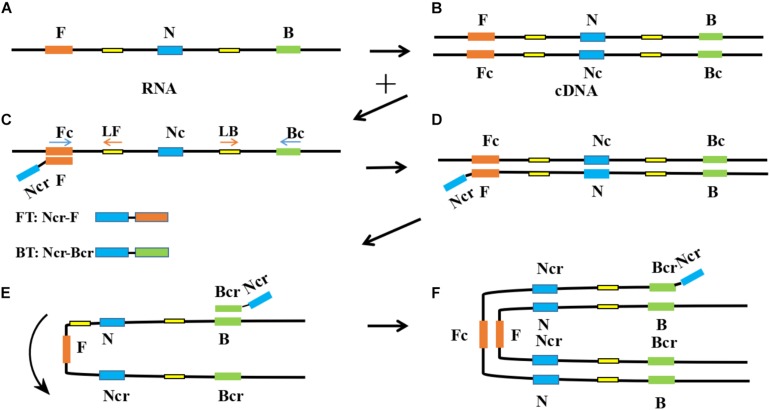
Schematic diagram of reverse transcription-polymerase spiral reaction (RT-PSR) amplification. **(A)** Template RNA; **(B)** Reverse transcription of template RNA into cDNA; **(C)** The F segment of Ft specifically recognizes and binds to the Fc segment on the target single-stranded cDNA; **(D)** Under the action of Bst DNA polymerase and dNTP, the primer extends to the 3’gap to form a double-stranded DNA structure; **(E)** The Nc segment on the newly formed single-stranded DNA rotate and combine with the N segment to form a U-shaped structure; **(F)** The Bcr segment of Bt is combined with the B segment of the U-shaped structure and extends around the structure.

**FIGURE 2:**
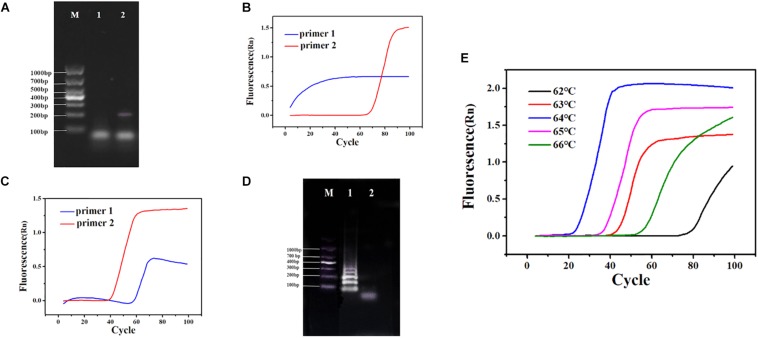
**(A)** PCR using cDNA as a template. Lane M: DL 1,000 DNA marker. (1) Negative control (NC), diethylpyrocarbonate (DEPC)-treated water; (2) 100 ng/μl cDNA. **(B)** Polymerase spiral reaction (PSR) fluorescence amplification curve of two sets of primers. **(C)** Reverse transcription (RT)-PSR fluorescence amplification curve of two sets of primers. **(D)** One percent agarose gel electrophoresis of RT-PSR amplified products of primer 2. Lane M: DL 1,000 DNA marker. (1) Positive sample; (2) NC, DEPC-treated water. **(E)** Fluorescence amplification curves of RT-PSR at different temperatures.

In RT-PSR, under the action of reverse transcriptase, viral RNA was reverse transcribed into cDNA. After that, the primers specifically bound to single-stranded cDNA and were extended by DNA polymerase. During amplification, the reverse transcription process continued, which greatly improved the amplification efficiency (Figure 1). After using the software to design two sets of primers (Supplementary Figure 1) and synthesize them, the next step was to establish the reaction process and select the best primers. Template RNA was reverse transcribed into cDNA and was amplified using the designed PCR primers to ensure availability for PSR. The target band was observed in the electrophoretogram of the PCR results, demonstrating availability of the cDNA template (Figure 2A). When cDNA was used as a template, primer 1 showed non-specific amplification, while amplification of primer 2 was performed normally (Figure 2B). When RNA was used as a template, primer 1 still showed dimerization and the amplification efficiency was significantly lower than that of primer 2 (Figure 2C). Therefore, primer 2 was selected as the best RT-PSR primer for CVA-16 detection. Subsequently, we used primer 2 for RT-PSR. CVA-16 genomic RNA (100 ng/μl) was used as a positive sample, while DEPC-treated water was used as NC, and the reaction products were separated by electrophoresis using a 1% agarose gel, which showed a characteristic DNA ladder in positive samples, while the NC showed no fragmentation (Figure 2D). This proved that under isothermal conditions, the method developed in this study performs reverse transcription and nucleic acid amplification in the same system. To achieve the best reaction performance, using CVA-16 genomic RNA (100 ng/μl) optimized some important factors, including optimal primers, reaction time, and temperature. According to the optimal primer screening, a series of temperature gradients (62–66°C) was set to observe the effects of different temperatures on the reaction, with 64°C being chosen as the optimum temperature of detection because it showed the shortest peak time and amplification efficiency was higher than at other temperatures (Figure 2E). The fluorescence curve had a longer plateau period, so the number of cycles was reduced to 80. Moreover, by comparing negative and positive results, adding 2 μl HNB dye with a mass fraction of 2% to 25 μl mixtures better distinguished the negative and positive samples (Supplementary Figure 2). Additionally, to avoid aerosol contamination and cross-contamination caused by nucleic acid amplification, we premixed and aliquoted the reaction solution. We added 10 μl paraffin oil to each tube and stored it at −20°C (Supplementary Figure 3).

### Sensitivity and Specificity

**FIGURE 3:**
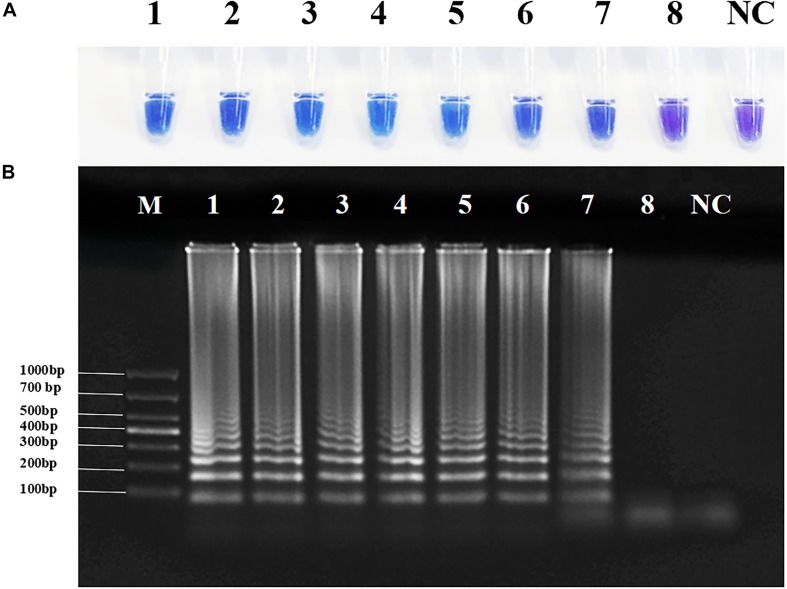
Sensitivity of reverse transcription-polymerase spiral reaction (RT-PSR) for Coxsackievirus A16 (CVA-16) detection. **(A)** The sensitivity test result of RT-PSR uses hydroxy naphthol blue (HNB) indicator; (1) 2.4 × 10^8^ copies/μL; (2) 2.4 × 10^7^ copies/μL; (3) 2.4 × 10^6^ copies/μL; (4) 2.4 × 10^5^ copies/μL; (5) 2.4 × 10^4^ copies/μL; (6) 2.4 × 10^3^ copies/μL; (7) 2.4 × 10^2^ copies/μL; (8) 2.4 × 10^1^ copies/μL; negative control (NC), diethylpyrocarbonate (DEPC)-treated water. **(B)** The sensitivity test result of RT-PSR using agarose gel electrophoresis. Lane M: DL 1,000 DNA marker. (1) 2.4 × 10^8^ copies/μL; (2) 2.4 × 10^7^ copies/μL; (3) 2.4 × 10^6^ copies/μL; (4) 2.4 × 10^5^ copies/μL; (5) 2.4 × 10^4^ copies/μL; (6) 2.4 × 10^3^ copies/μL; (7) 2.4 × 10^2^ copies/μL; (8) 2.4 × 10^1^ copies/μL; negative control (NC), diethylpyrocarbonate (DEPC)-treated water.

To determine the sensitivity of RT-PSR, we tested gradient-diluted plasmid templates. Under natural light, when the target template concentration was ≥ 2.4 × 10^2^ copies/μl, the color of the reaction solution became sky blue. When the template was DEPC-treated water or the template concentration was 2.4 × 10^1^ copies/μl, the color of the reaction mixture was violet. Agarose gel electrophoresis yielded the same results (Figure 3). Amplification curves were observed when the template concentration was ≥ 2.4 × 10^2^ copies/μl. When the template concentration was 2.4 × 10^1^ copies/μl or the template was DEPC-treated water, no change in fluorescence were observed (Supplementary Figure 4a). Therefore, the detection limit of this method was between 2.4 × 10^2^ and 2.4 × 10^1^ copies/μl, while the detection limit of qRT-PCR was between 2.4 × 10^3^ and 2.4 × 10^2^ copies/μl (Supplementary Figure 5).

**FIGURE 4:**
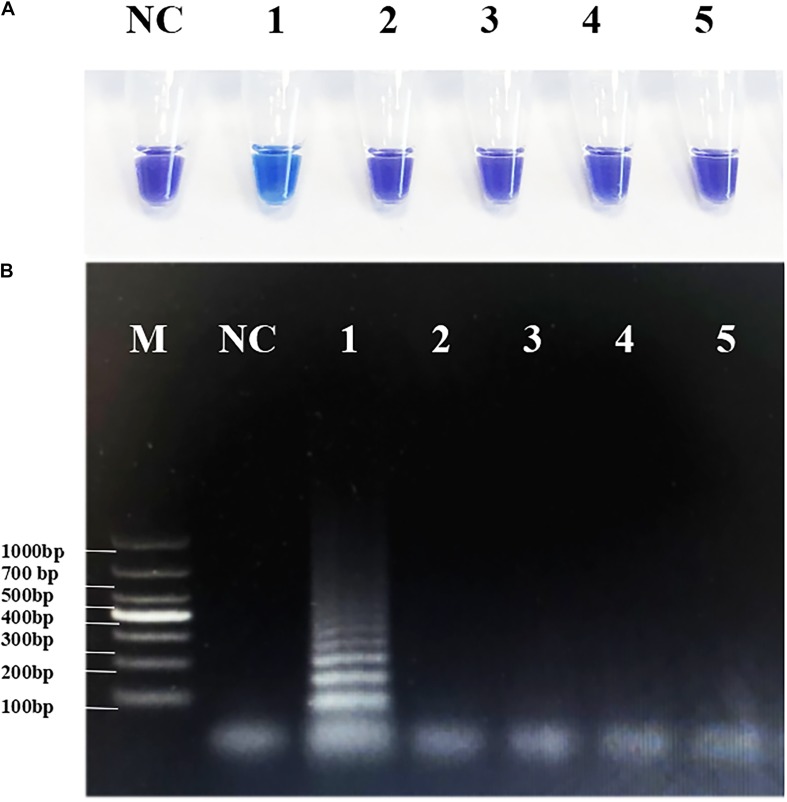
Specificity of the reverse transcription-polymerase spiral reaction (RT-PSR) method. **(A)** The specificity test result of RT-PSR uses hydroxy naphthol blue (HNB) indicator; (1) Coxsackievirus A16 (CVA-16); (2) Norwalk virus genogroup II (NVG II); (3) Rotavirus A (RVA); (4) CVA-6; (5) Enterovirus (EV)-A71; negative control (NC), diethylpyrocarbonate (DEPC)-treated water. **(B)** The specificity test result of RT-PSR using agarose gel electrophoresis. Lane M: DL 1,000 DNA marker. (1) Coxsackievirus A16 (CVA-16); (2) Norwalk virus genogroup II (NVG II); (3) Rotavirus A (RVA); (4) CVA-6; (5) Enterovirus (EV)-A71; negative control (NC), diethylpyrocarbonate (DEPC)-treated water.

To avoid false-positive errors in RT-PSR, we selected several common viruses with high infection rates in children for specific testing. Through the clinical diagnostic standard test, all four non-target viruses had positive clinical test results, so they can be used for specific detection (Supplementary Figure 6). The RNA templates of NVG II, RVA, CVA-6, and EV-A71 were tested to determine the specificity of RT-PSR. The results for CVA-16 were sky blue, and the rest were violet. Consistent with agarose electrophoresis results, none of the above viruses showed cross-reactivity (Figure 4), and the same result was shown by the fluorescence curve (Supplementary Figure 4b), which indicated that the system established in this study had good specificity.

### Clinical Specimens

Large amounts of virus are present in the stools of HFMD patients. After testing 40 clinical samples by the two methods, 25 cases were positive and 15 were negative by both methods (Table 2). The coincidence rate of the two methods was 100%. Thus, this illustrated that the method was sensitive and reliable and has the potential to be applied to the screening of clinical CVA-16 samples. Through the collation and analysis of the epidemiological situation, we found that the age of the 40 participants ranged from 0.5 to 10 years, with an average of 3.19 years, and 45% were boys. Ninety-five percent of the children had clinical symptoms of hand, foot, and skin rash and oral herpes (Supplementary Table 1). Most children also had fever and inflammation of other tissue. Depending on the symptoms and severity of the patient’s condition, after symptomatic treatment with antiviral and anti-inflammatory drugs such as interferon, andrographis paniculata, the overall prognosis of the patient is good.

**TABLE 2:** Comparison of qRT-PCR with reverse transcription-polymerase spiral reaction (RT-PSR) results.

Sample no.	Relative quantification (copies/μl)	RT-PSR	Sample no.	Relative quantification (copies/μl)	RT-PSR
1	1.25 × 10^5^	+	21	5.6 × 10^5^	+
2	5.6 × 10^5^	+	22	5.6 × 10^5^	+
3	5.6 × 10^5^	+	23	5.6 × 10^5^	+
4	6.2 × 10^6^	+	24	2.3 × 10^3^	+
5	5.98 × 10^6^	+	25	4.0 × 10^3^	+
6	1.57 × 10^5^	+	26	0	−
7	3.55 × 10^4^	+	27	0	−
8	6.5 × 10^4^	+	28	0	−
9	4.0 × 10^3^	+	29	0	−
10	6.5 × 10^3^	+	30	0	−
11	4.0 × 10^4^	+	31	0	−
12	2.8 × 10^3^	+	32	0	−
13	5.6 × 10^5^	+	33	0	−
14	6.2 × 10^6^	+	34	0	−
15	5.6 × 10^5^	+	35	0	−
16	6.2 × 10^6^	+	36	0	−
17	6.2 × 10^6^	+	37	0	−
18	1.72 × 10^6^	+	38	0	−
19	4.0 × 10^3^	+	39	0	−
20	5.6 × 10^3^	+	40	0	−

## Discussion

Hand, foot, and mouth disease is a common childhood disease that is increasingly recognized as a major health problem, causing a huge economic and health burden in China (Zheng et al., 2017). In China, > 2 million cases of HFMD were recorded by the Chinese Ministry of Health in 2015 (Huang et al., 2018). The first recorded association of HFMD with a specific virus was with CVA-16 in 1959 in Birmingham, AL, United States (Alsop et al., 1960). Although CVA-16 does not tend to cause neurological disease during infections, the virus can induce erythematous, enanthem of the oral cavity, and acute viral cardiomyopathy (Koh et al., 2016). Emerging HFMD has a huge impact on the medical system worldwide. For example, where children are gathered in kindergartens, the spread of the virus increases rapidly (Xu et al., 2012; Rao et al., 2013). By observing the clinical data of 40 cases in this study, we found that the incidence of HFMD was highest in children aged < 5 years. This may have been due to the lower immunity of infants and young children, and hand–mouth behavior of children is more frequent, making it easier for the virus to spread. The clinical symptoms and signs are more typical, and the prognosis is better after treatment. Therefore, health education on HFMD prevention and control and awareness of HFMD should be enhanced. In addition, we must strengthen surveillance of the epidemic and detect patients as early as possible. The capability for accurate diagnosis is a prerequisite for adequate clinical and public health response, and early detection and proper treatment remain the keys to reducing mortality (Li et al., 2018). To control widespread concern, developing rapid detection methods for HFMD has become a focus in some countries.

In this study, we developed a method based on RT-PSR for rapid identification of CVA-16. In comparison with traditional approaches and other molecular biological methods for CVA-16 detection, RT-PSR was superior. Firstly, compared with traditional virus isolation and identification (Lin et al., 2008), our method greatly reduces the time required and is more convenient to operate. Secondly, compared with the LAMP method (Ding et al., 2014), the primer design and amplification principle of RT-PSR are simpler. Only a pair of main primers and a pair of accelerated primers can amplify a large amount of template RNA in a short time. Thirdly, using the assay developed in this study, the detection limit of this method can reach between 2.4 × 10^2^ and 2.4 × 10^1^ copies/μl, which is about 10 times more sensitive than qRT-PCR. Our method greatly shortens the clinical testing time while avoiding the use of large PCR instruments. Finally, the reaction can achieve the purpose of visual detection without the use of fluorescent probes, which effectively reduces the cost. The operator only needs to add the enzyme and RNA template, and briefly centrifuge before the reaction, which further simplifies the operation. The method provides feasible technical support for real-time and on-site testing, especially in poorly resourced rural and suburban areas, which will help expand the control of HFMD and other viral diseases.

## Conclusion

We developed a novel approach for rapid detection of CVA-16. The entire process, including reverse transcription and nucleic acid amplification, can be completed in a single tube within 40 min. Without using any sophisticated instruments, the detection limit of RT-PSR can reach between 2.4 × 10^2^ and 2.4 × 10^1^ copies/μl, with excellent specificity. The method was validated by clinical specimens and proven to be highly efficient and suitable for detecting CVA-16. In the future, we envision that RT-PSR may demonstrate even greater potency in clinical applications and become a novel method for isothermal nucleic acid testing of other single-stranded RNA viruses.

## Data Availability Statement

All datasets generated for this study are included in the article/Supplementary Material.

## Ethics Statement

The studies involving human participants were reviewed and approved by Ethics Committee of the Changchun Children’s Hospital. Written informed consent to participate in this study was provided by the participants’ legal guardian/next of kin.

## Author Contributions

XS, HL, and JL conceived and designed the experiments. SH, YH, YZ, and HY performed the experiments. LW, LS, and JW contributed to the reagents and materials. SH, BP, and XS analyzed the data and wrote the manuscript. All authors have contributed and approved the final version of the manuscript.

## Conflict of Interest

The authors declare that the research was conducted in the absence of any commercial or financial relationships that could be construed as a potential conflict of interest.
