# Supplementary material for: A Reverse Transcription-Polymerase Spiral Reaction (RT-PSR)-Based Rapid Coxsackievirus A16 Detection Method and Its Application in the Clinical Diagnosis of Hand, Foot, and Mouth Disease
Source: Front Microbiol. 2020 May 12;11:734. doi: 10.3389/fmicb.2020.00734 (PMC7236501; doi:10.3389/fmicb.2020.00734)
Supplement: Supplementary file 1 [file Data_Sheet_1.docx]

Supplementary material

**A reverse transcription-polymerase spiral reaction (RT-PSR)-based rapid Coxsackievirus A16 detection method and its application in the clinical diagnosis of Hand, foot, and mouth disease**

Shiyu He^1^ , Yanzhi Huang^2^ , Yanling Zhao^2^ , Bo pang^1^, Lixue Wang^2^, LiweiSun^2^, Haoyan Yu^1^, Juan wang^1^, Juan Li^1^, Xiuling Song^1^, *, Hui Li^1^, *

^1^Department of Hygienic Inspection, School of Public Health, Jilin University, 1163 Xinmin Street, Changchun 130021, Jilin, China

^2^Research Laboratory, Changchun Children's Hospital, Changchun130061, Jilin, China

***Corresponding author. E-mail address:** songxiuling@jlu.edu.cn & leehui@jlu.edu.cn

**Supplementary Figures**

**
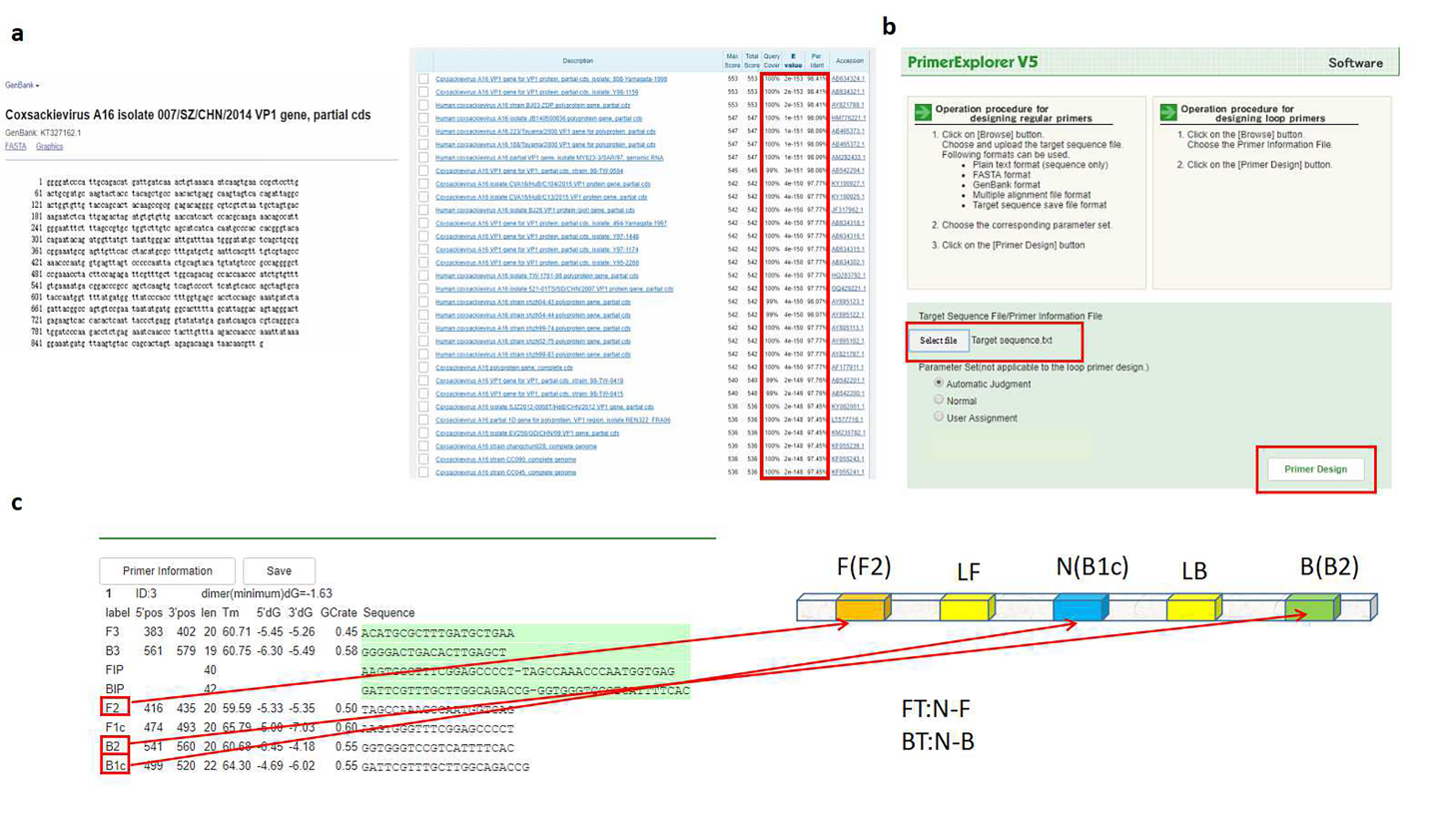

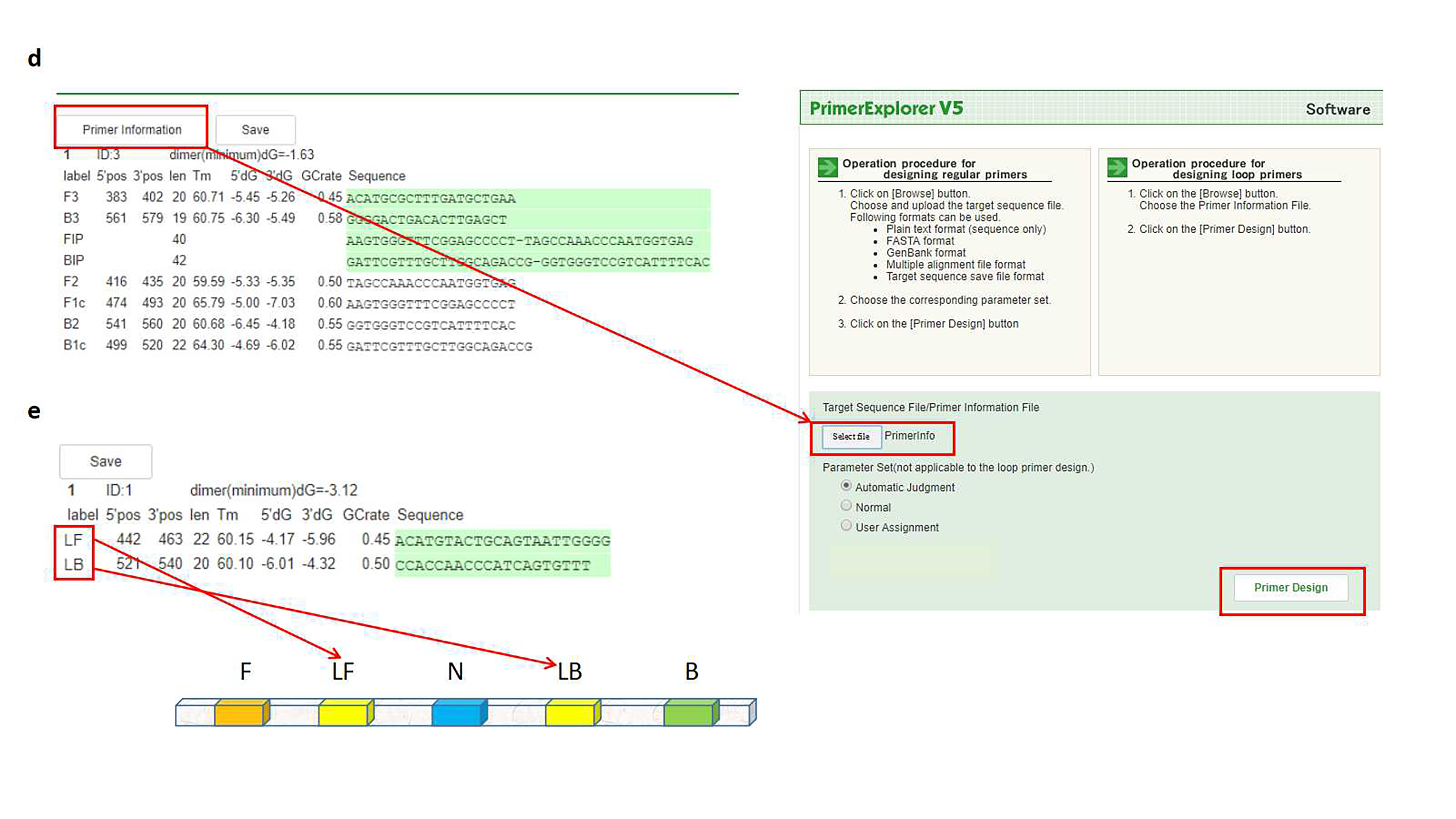
**

**Supplementary Figure 1: a** Finding and blasting align target sequences in NCBI; **b** Design and selection of primer fragments using software (primer explorer V5); **c** Construction of the primary primer; **d** Save primary primer information and use primer explorer V5 again to design accelerate primer; **e** Construction of the accelerate primer.


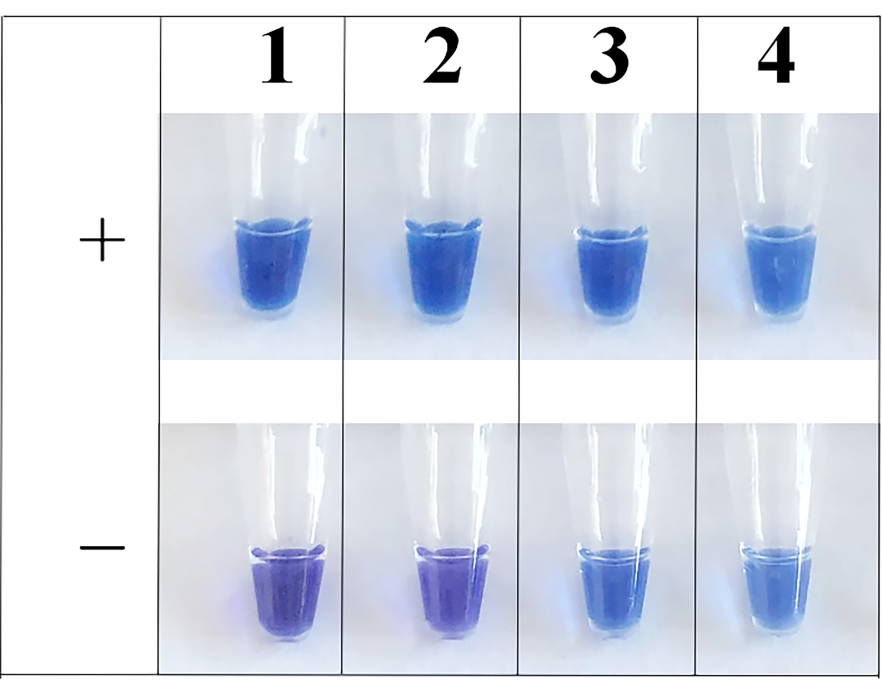


**Supplementary Figure 2:** Investigation on the optimal concentration of HNB for RT-PSR. group1-4: 2.5µLHNB, 2.0µLHNB, 1.5µLHNB, 1.0µLHNB;＋: Positive sample; －: Negative sample

**
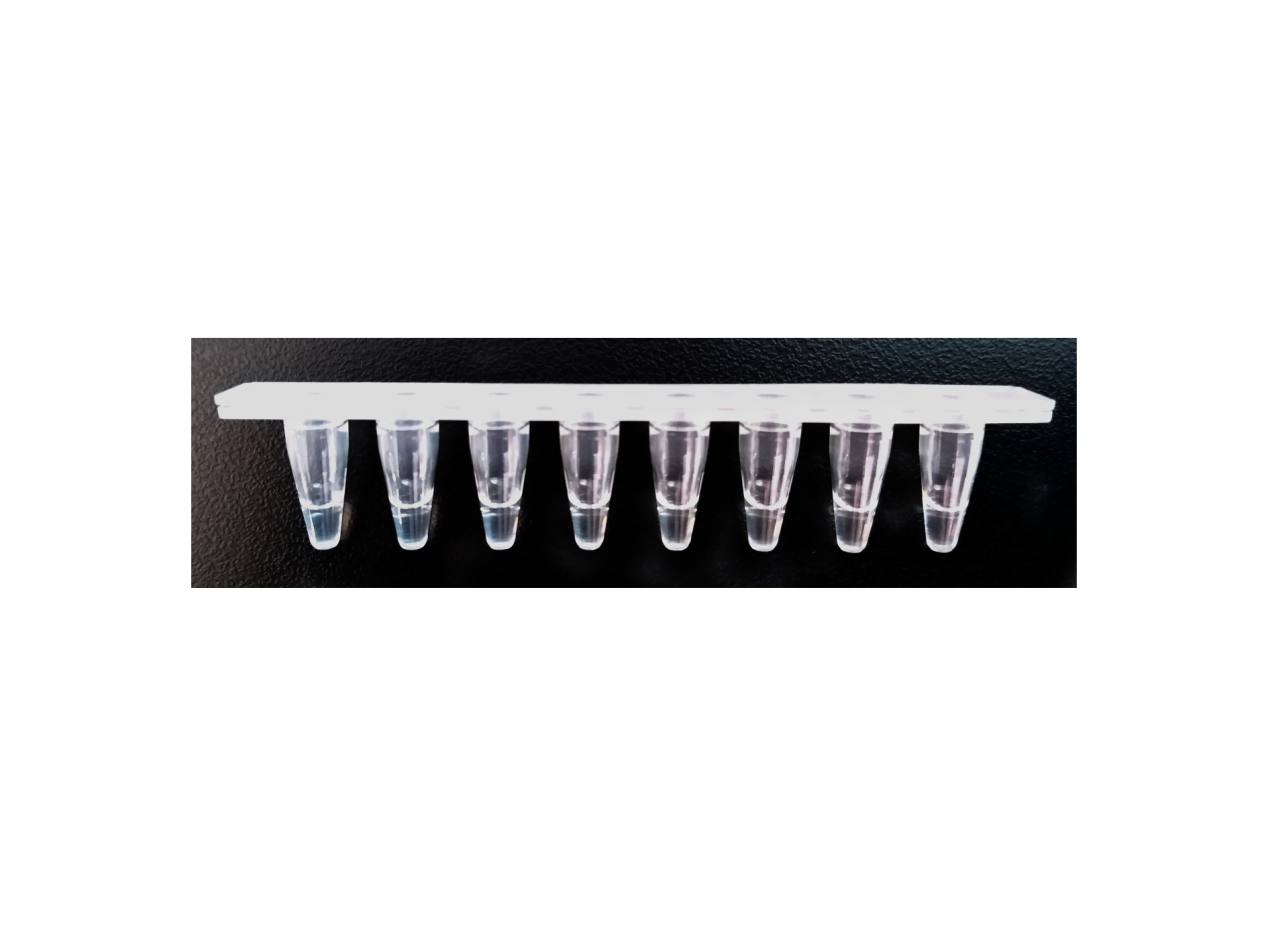
**

**Supplementary Figure 3:** RT-PSR master mix with paraffin oil seal.


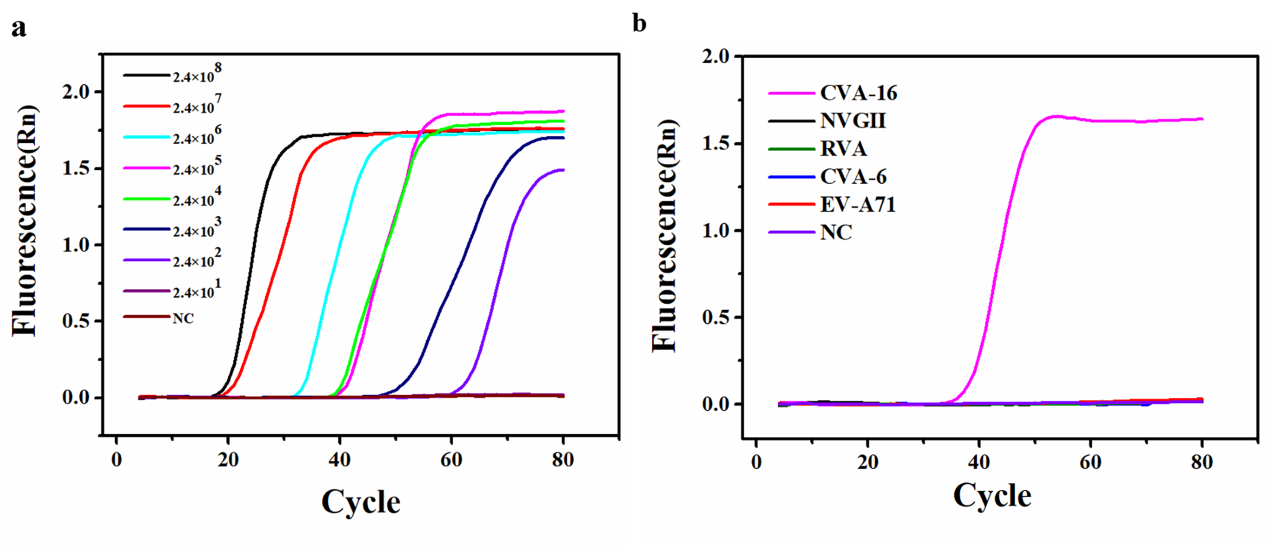


**Supplementary Figure 4:** a Sensitivity detection fluorescence curve of RT-PSR; b Specific detection fluorescence curve.of RT-PSR.


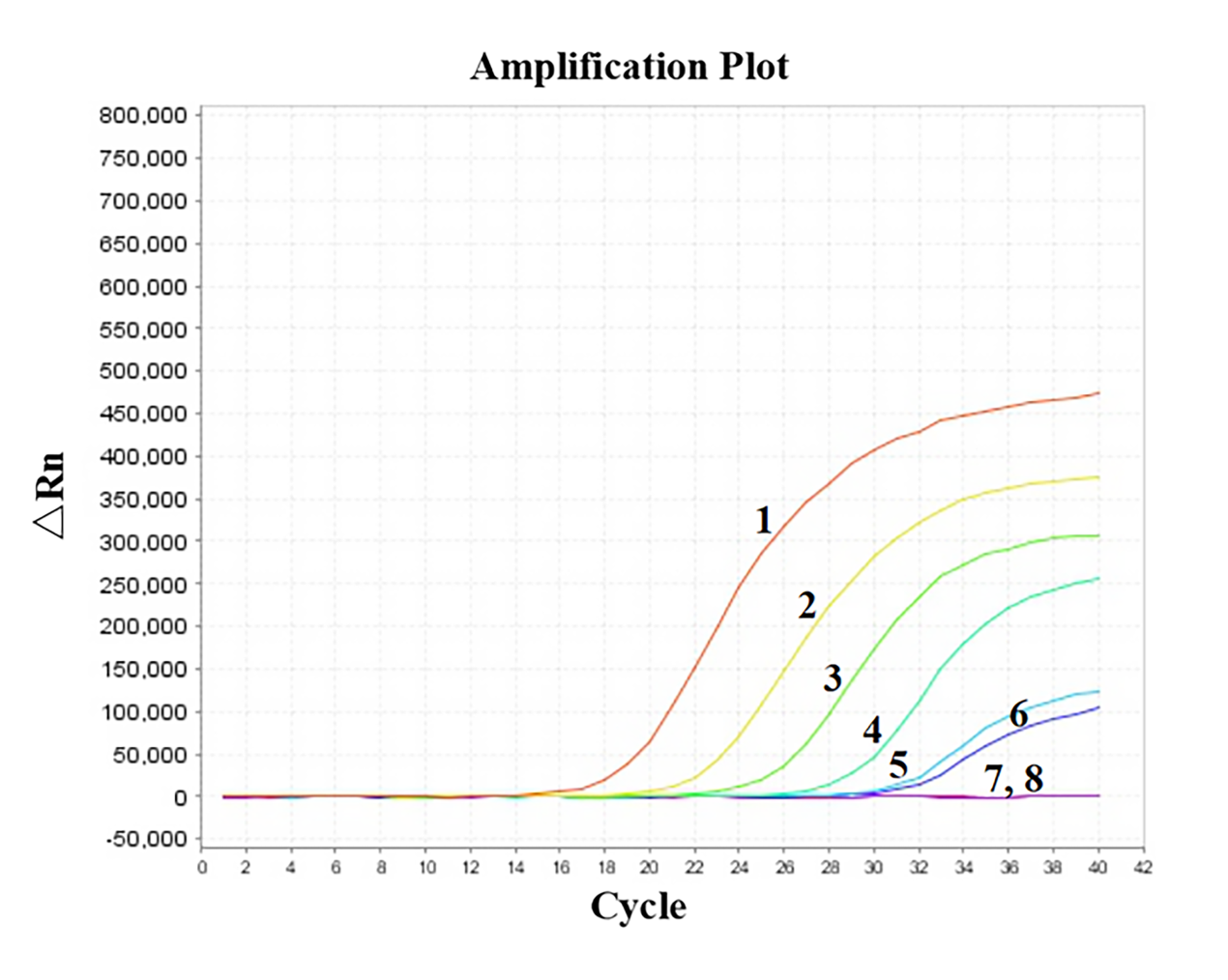


**Supplementary Figure 5:** Sensitivity of quantitative real-time PCR for for CVA-16 detection. 1, 2.4×10^8^ copies/µL, 2, 2.4×10^7^ copies/µL, 3, 2.4×10^6^ copies/µL, 4, 2.4×10^5^ copies/µL, 5, 2.4×10^4^ copies/µL, 6, 2.4×10^3^ copies/µL, 7, 2.4×10^2^ copies/µL, 8, 2.4×10^1^ copies/µL.


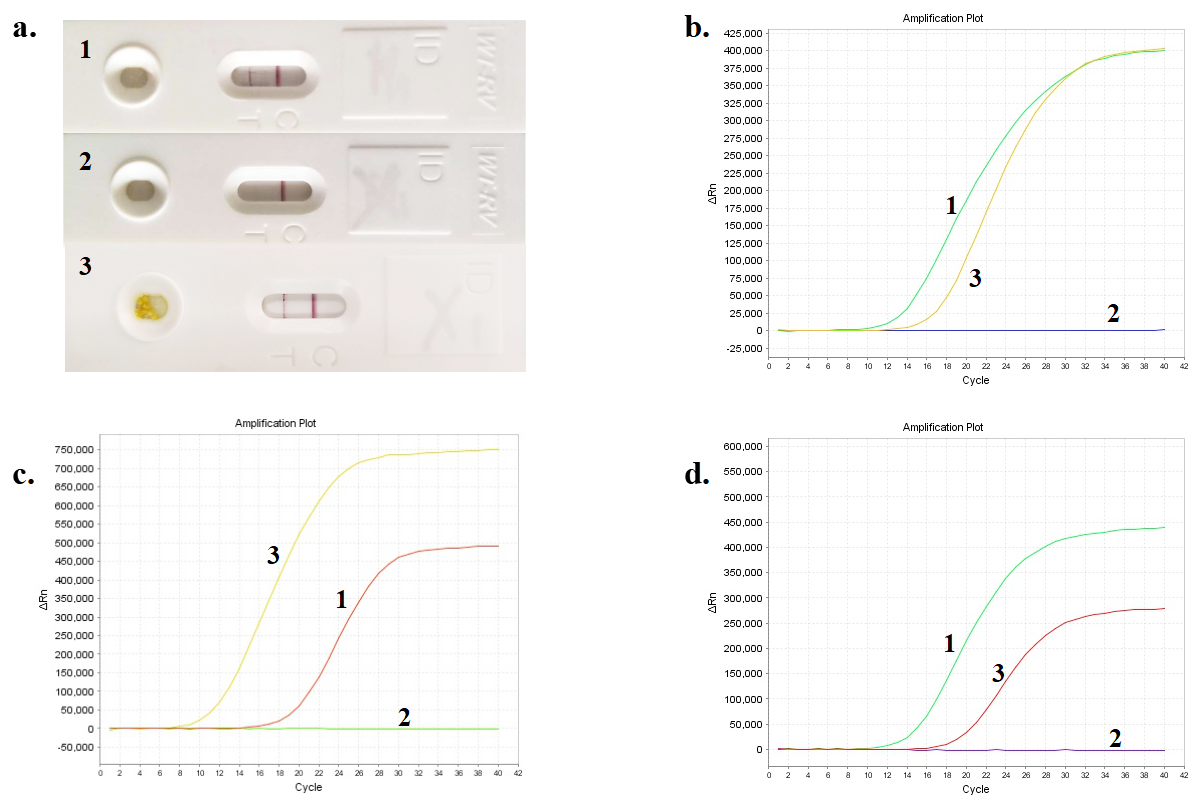


**Supplementary Figure 6:** Non-target virus clinical standardized test results. a Detection results of RVA using colloidal gold clinical diagnostic kit; 1, positive control, 2, negative control, 3, RVA sample; b qRT-PCR fluorescent probe test kit for EV-A71 detection results; 1, positive control, 2, negative control, 3, EV-A71 sample; c qRT-PCR fluorescent probe test kit for CVA-6 detection results; 1, positive control, 2, negative control, 3, CVA-6 sample; d qRT-PCR fluorescent probe test kit for NVG II detection results; 1, positive control, 2, negative control, 3,NVG II sample.

**Supplementary Table 1** Basic epidemiological information of clinical cases

| **Characteristics** | **Positive sample** | **Negative**  **sample** | **total** |
| --- | --- | --- | --- |
| Number of pants,(%) | 25 (62.5) | 15 (37.5) | 40 |
| Age, year | 3.54±2.16 | 2.61±1.41 | 3.19±1.95 |
| Sex,(%) |  |  |  |
| male | 12 (48.00) | 6 (40.00) | 18(45.00) |
| female | 13 (52.00) | 9 (60.00) | 22(55.00) |
| days in hospital | 6.04±1.63 | 7.60±4.78 | 6.63±3.14 |
| Virus typing,(%) |  |  |  |
| CVA-16+ | 25 (100.00) | 0（0） | 25(62.50) |
| CVA-6+ | 0（0） | 7 (47.67) | 7(17.50) |
| EV-A71+ | 0（0） | 6 (40.00) | 6(15.00) |
| negative | 0（0） | 2 (13.33) | 2(5.00) |
| days between symptom onset and sample collection | 2.64±1.11 | 3.20±1.52 | 2.85±1.29 |
| Rash, oral herpes |  |  |  |
| yes | 25(100.00) | 13(86.67) | 38(95.00) |
| no | 0(0.00) | 2(13.33) | 0(5.00) |
| fever |  |  |  |
| yes | 16 (64.00) | 10 (66.67) | 26(65.00) |
| no | 9 (36.00) | 5 (33.33) | 14(35.00) |
| Other inflammation |  |  |  |
| yes | 17(68.00) | 11(73.33) | 28(70.00) |
| no | 8(32.00) | 4(26.67) | 12(30.00) |
| Antiviral therapy |  |  |  |
| yes | 12 (48.00) | 9 (60.00) | 21(52.50) |
| no | 13 (52.00) | 6 (40.00) | 19(47.50) |
| Prognosis |  |  |  |
| cure | 25(100.00) | 11(73.33) | 36(90.00) |
| Improve | 0(0.00) | 4(26.67) | 4(10.00) |
